# Trait and state-related characteristics of thalamo-cortical circuit disruption in bipolar disorder: a prospective cross-sectional study

**DOI:** 10.3389/fpsyt.2023.1067819

**Published:** 2023-05-26

**Authors:** Can Zeng, SuQun Liao, Weidan Pu

**Affiliations:** ^1^Department of Psychology, Shaoguan University, Shaoguan, China; ^2^Department of Clinical Psychology, The Third Xiangya Hospital, Central South University, Changsha, China

**Keywords:** bipolar depression, bipolar remission, neuroImage, thalamus, resting state

## Abstract

**Objective:**

The purpose of this study is to investigate the shared and distinct thalamic-cortical circuit between bipolar depression and remission, as well as to investigate the trait and state-related characteristics of the abnormal thalamic-cortical circuit in bipolar disorder.

**Methods:**

Resting-state functional magnetic resonance imaging was performed on 38 bipolar depression patients, 40 bipolar remission patients, and 39 gender-matched healthy controls (rsfMRI). The thalamic subregions were used as seed points to draw the functional connectivity of the entire brain, and then the shared and distinct thalamic-cortical circuits between bipolar depression and remission were compared.

**Results:**

When compared to the healthy group, both groups of patients had significantly lower functional connectivity between the rostral temporal thalamus and the lingual gyrus, the posterior parietal thalamus, the precuneus/cerebellum, and the occipital thalamus and the precuneus; however, functional connectivity between the premotor thalamus and the superior medial frontal was significantly lower in depression.

**Conclusion:**

This study discovered that both bipolar depression and remission had abnormal sensorimotor-thalamic functional connectivity, implying that it is a trait-related characteristic of bipolar disorder; however, the decline in prefrontal-thalamic connectivity exists specifically in bipolar depression, implying that it is a state-related characteristic of bipolar disorder.

## 1. Introduction

Bipolar disorder (BD) is a severe mental disease characterized by recurrent depression and mania or hypomania (1). According to neuroimaging studies, BD patient’s cognition and emotion-related brain regions are abnormally activated during mood episodes and remission periods (2), although the unusual activation is discrepant during various periods; therefore, probing for shared and distinction of brain activities in different states is of great importance to acquire the trait and state-related characteristics of bipolar disorder, which may help to improve the precise diagnosis and treatment for this severe mental disorder.

Previous researches on BD found abnormal brain activity largely located in the prefronto-limbic system, especially in the amygdala-prefrontal circuit and prefronto-striatal circuit (3, 4). Vargas et al. (3) conducted a comprehensive review of eight studies, comparing the resting state fMRI of 241 BD patients and 278 healthy controls, and discovered prefronto-limbic cortex connectivity disruption in BD compared to healthy controls. And researches on bipolar depression and remission, found abnormal activation primarily involved in the prefrontal and limbic cortex as well (5). Most researchers applied the amygdala as a seed point to map the cortical-amygdala connectivity during tasks related with emotional processing, and discovered that in both states, the amygdala and the prefrontal cortex often emerged abnormal activation (6–9) and connectivity (10, 11). In addition, during tasks involved in rewarding processing, abnormal activation and functional connectivity were consistently observed in the prefronto-striatal circuit in bipolar depression or remission episode (12, 13). In summary, prior research has established that individuals with bipolar disorder exhibit comparable irregularities in the prefronto-limbic system during both depression and remission phases. However, there is a lack of direct comparison to identify shared and distinctive biomarkers between these states. Furthermore, prior investigations have primarily focused on the amygdala and striatum within the limbic system, neglecting the crucial role of the thalamus in the development of bipolar disorder.

The thalamus plays a vital role as an information transfer relay station that receives all sensory information, excluding those from the olfactory region. Afferent or efferent information after being transferred in the thalamus are then paralleled in projection to different brain regions; thus, the thalamus stands in the center of bottom-up transmission and top-down regulation of the brain, plays a vital role in emotion and cognition regulation (14). Thalamic dysfunction is a well-established characteristic of schizophrenia. Although, bipolar disorder is considered to be within the same spectrum as schizophrenia (15), the studies investigating thalamic abnormalities in bipolar disorder have been limited. Only a few studies have explored this area of research, including findings of abnormalities of Steullet (16) in the thalamic reticular nucleus in bipolar disorder patients. Hiber et al. (17) also reported reduced thalamic volume, but no significant difference between bipolar type I and type II. Furthermore, research based on functional analysis revealed hypoconnectivity between the thalamus and the prefrontal cortex (18), and hyperconnectivity between the sensorimotor cortex and thalamus (15).

Thus far, researchers have not extensively investigated thalamic abnormalities in different states of bipolar disorder, which makes it challenging to distinguish the state and trait characteristics of bipolar disorder. The limited comparative studies that have focused on bipolar depression and remission include comparison of the differences between the two states using the whole-brain functional connectivity method in a resting state by Lv et al. (11), and comparison of brain activation between the two groups using the Stroop task (12) by Kronhaus et al. (9). However, both studies have neglected to examine the thalamus.

The thalamus comprises several diverse nuclei that project to specific regions of the cortex. For instance, the dorsomedial nucleus (MD) projects to the prefrontal cortex, while the lateral geniculate nucleus projects to the occipital cortex (19). Therefore, by mapping the functional connectivity anomalies between the thalamus subregions and the cortex, may help us more accurately understand the shared and distinction of brain functions in the two states. In this study, we applied the thalamus subregions atlas from Human Brainnetome Atlas,^1^ which parcellated the thalamus into eight subdivisions bilaterally based upon the current largest database of brain activation (20), and it has been used in our past studies as well (21).

## 2. Methods

### 2.1. Subjects

A total of 117 subjects participated in this study, including 38 bipolar depression patients, 40 bipolar remission patients, and 39 healthy controls. All patients diagnosed with depression were experiencing an episode, while the patients in remission were diagnosed as being in remission for a minimum of 3 months by psychiatrist. The inclusion criteria of the two groups include: (1) age between 18 and 60 years, (2) received at least 9 years of school education, (3) right-handedness, (4) met the criteria of structured clinical patient interview version (SCID-I/P) for BD, (5) for the bipolar depression group the of Hamilton Depression Scale score ≥ 17 points, and Young’s Mania Scale score ≤ 6 points, and (6) for the bipolar remission group the Hamilton Depression Scale score < 17 points, and the Yang’s Mania Scale score ≤ 6 points.

Healthy subjects were recruited from the population and evaluated using a structured clinical interview version (nonpatient version, SCID-I/NP) to ensure that they and their first-degree relatives were immune from any mental illness. The exclusion criteria both for patients and healthy were as follows: (1) having other major physical or mental diseases, intellectual disability, substance abuse (except tobacco), and personality disorders; (2) having used benzodiazepines or alcohol within 24 h before the two interviews or fMRI scan; (3) received electric shock treatment in the past. All the patients were recruited from Xiangya Second Hospital of Central South University, and interviewed by two psychiatrists at the hospital.

This study obtained ethical approval from the ethics committee of Xiangya Second Hospital and the study was conducted after obtaining informed consent from the subjects.

### 2.2. MRI data acquisition and preprocessing

Entire functional magnetic resonance imaging (fMRI) data were acquired using a Philips Gyroscan Achieva 3.0 T scanner [36 layers, matrix = 64 × 64, flip angle (FA) of 90^0^, echo time (TE) = 30 ms, repetition time (RT) = 2,000 ms, gap = 0 mm, slice thickness = 4 mm]. Each subject was scanned for 250 volumes, and the area of the brain was completely covered. During the scan, subjects were instructed to close their eyes and to keep quiet, and their head movements were restricted. The original data were preprocessed with DPARSF (22), and data were rejected when the head movement exceeded 3 mm, three subjects’ data were excluded for this reason.

To allow scanner calibration and subjects to adapt to the environment, the first 10 volumes were discarded, then the remaining 240 volumes were processed by SPM12.^2^ During preprocessing, the images were spatially normalized to the Montreal Neurological Institute (MNI) standard template and resampled to 4 mm × 4 mm × 4 mm voxels applying standard parameters. After normalization, the Blood Oxygenation Level Dependent (BOLD) signal was first detrended and then underwent a band-pass filter (0.01–0.08 Hz) to decrease high-frequency physiological noise and low-frequency drift artifacts. Nuisance covariates, with global mean signals, head motion parameters, cerebrospinal fluid signals, and white matter signals, were regressed out from the BOLD signals. Finally, considering the probable confounding effect of micromovements on neuroimaging data (23), the investigators also regressed the framewise displacement (FD) value during preprocessing.

### 2.3. Functional connectivity analysis

**Figure 1:**
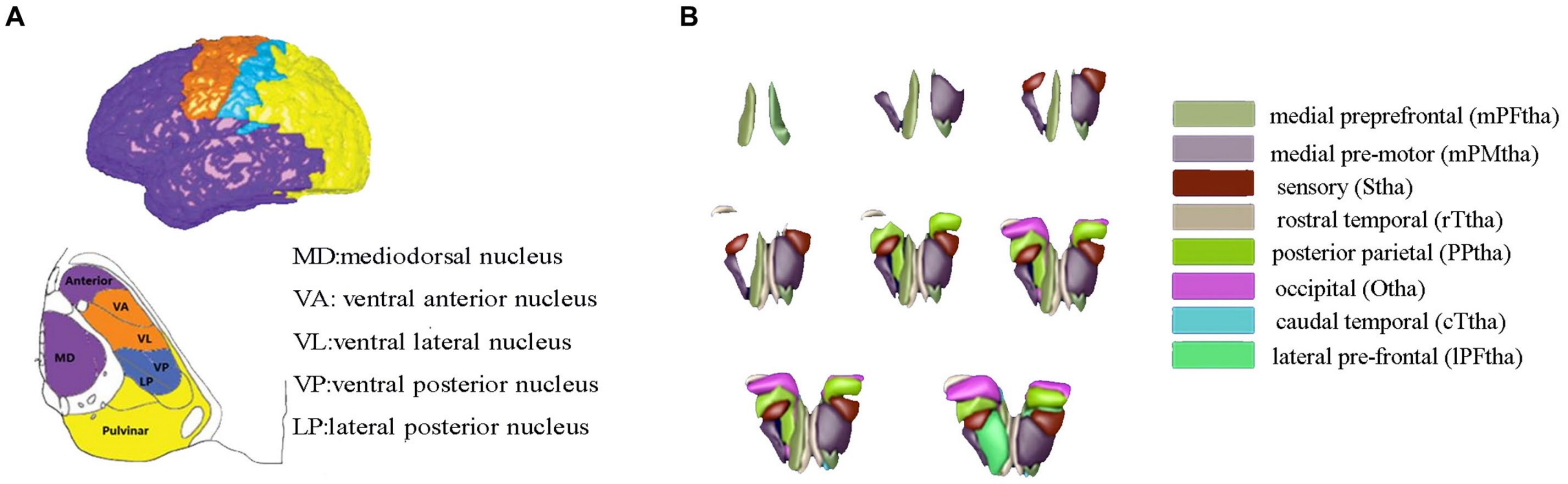
Thalamic segment atlas based on both the mode of the histological **(A)** and cortical-thalamic subregions connectivity **(B)**. Panel **(A)** based on histological atlas. Nuclei have been color-coded corresponding to the cortical zone, Panel **(B)** showed the 16 thalamic subregions from the human Brainnetome Atlas. The mPFtha is thought to include the ventral anterior (VA), the mediodorsal nucleus (MD), and parts of the anterior complex that project to the prefrontal cortices; The mPMtha is thought to include the ventral lateral nucleus (VL) and VA that project to the premotor cortices; The Stha is thought to include the lateral posterior nucleus (LP) and ventral posterior nucleus (VP), which project to the somatosensory cortices; The rTtha is thought to include parts of the anterior complex that projects to the limbic areas in the medial temporal lobe, and parts of MD which receives the inputs from the temporal lobe; The PPtha is thought to include the anterior parts of the pulvinar that projects to the posterior parietal cortices (PPC); The Otha is thought to include the lateral geniculate nucleus (LGN), parts of the inferior pulvinar, and some intralaminar nuclei projecting to the occipital cortices; The cTtha is thought to include the VL and VP which connect to the premotor and somatosensory cortices, and parts of the lateral and inferior pulvinar that project to the temporal lobe; The lPFtha is thought to include MD and parts of the anterior complex that project to prefrontal cortices.

The thalamus was classified into eight subregions bilaterally according to the Human Brainnetome Atlas, which are named as the medial prefrontal thalamus, medial premotor thalamus, sensory thalamus, rostral temporal thalamus, posterior parietal thalamus, occipital thalamus, caudal temporal thalamus, and lateral prefrontal thalamus, each thalamic subregion was carefully chosen as the region of interest (ROI) to map functional connectivity with the entire brain, and connectivity maps (*z* values) of each seed was calculated, accordingly (see Figure 1).

### 2.4. Statistical analysis

The data was further analyzed after the preprocessing. Firstly, SPM12 (see footnote 2) was applied to perform an ANOVA test across the three groups as to compare the difference in patterns of functional connectivity of each seed point with the rest of the brain (FWE correction, *p* < 0.05), a strict voxel-wise threshold was set for controlling the problem of multiple comparisons, as statistician performed 16 ANOVAs for each thalamic subregion. Then, the selection of the significant clusters in ANOVA as the masks and further the *post hoc t*-test were performed (*p* < 0.05 corrected by FWE). At last, we analyzed the correlation between the mean value of cortical-thalamic connectivity that appeared in intergroup differences and the clinical variables score of the Hamilton depression scale.

## 3. Results

### 3.1. Participant demographics

The demographic and clinical data of the subjects are given in Table 1. The two patients’ groups showed a significant difference in HAMD scores, the depressive group was significantly higher than the remission, and no other significant difference was seen (Table 1).

**Table 1:** Demographic and clinical characteristics.

Parameter	BDD	BDR	HC	ANOVA		BDD *Vs* BDR	
	*n* = 38	*n* = 40	*n* = 39	*F*	*p* (two-tailed)	*t*/*χ*^2^	*p* (two-tailed)
	M(SD)	M(SD)	M(SD)				
Age	26.47 (6.89)	26.72 (7.03)	25.28 (5.4)	0.553	0.557	-	-
Education (years)	13.56 (2.80)	12.97 (2.74)	13.74 (2.45)	0.899	0.41	-	-
Sex (male/female)	15/23	21/19	23/16	3.435	0.180	-	-
HAMD scores	21.1 (4.39)	4.12 (3.63)	-	-	-	18.73	0.00^**^
YMRS scores	2.05 (2.01)	2.35 (3.04)	-	-	-	0.51	0.614
Onset age	21.8 (5.07)	22.17 (5.95)	-	-	-	0.86	0.357
Duration (years)	56.2 (61.39)	49.43 (47)	-	-	-	2.87	0.094
Medication (years)	6.94 (16.13)	13.75 (29.58)	-	-	-	1.25	0.214
Antidepressant (*n*)	18/38	8/40	-	-	-		
Antipsychotic (*n*)	28/38	23/40	-	-	-		

BDD, bipolar depression; BDR, bipolar remission; HC, healthy control; HAMD, Hamilton depression scale; and YMRS, Young’s Mania scale.

### 3.2. Cortical-thalamic connectivity analysis across three groups

**Figure 2:**
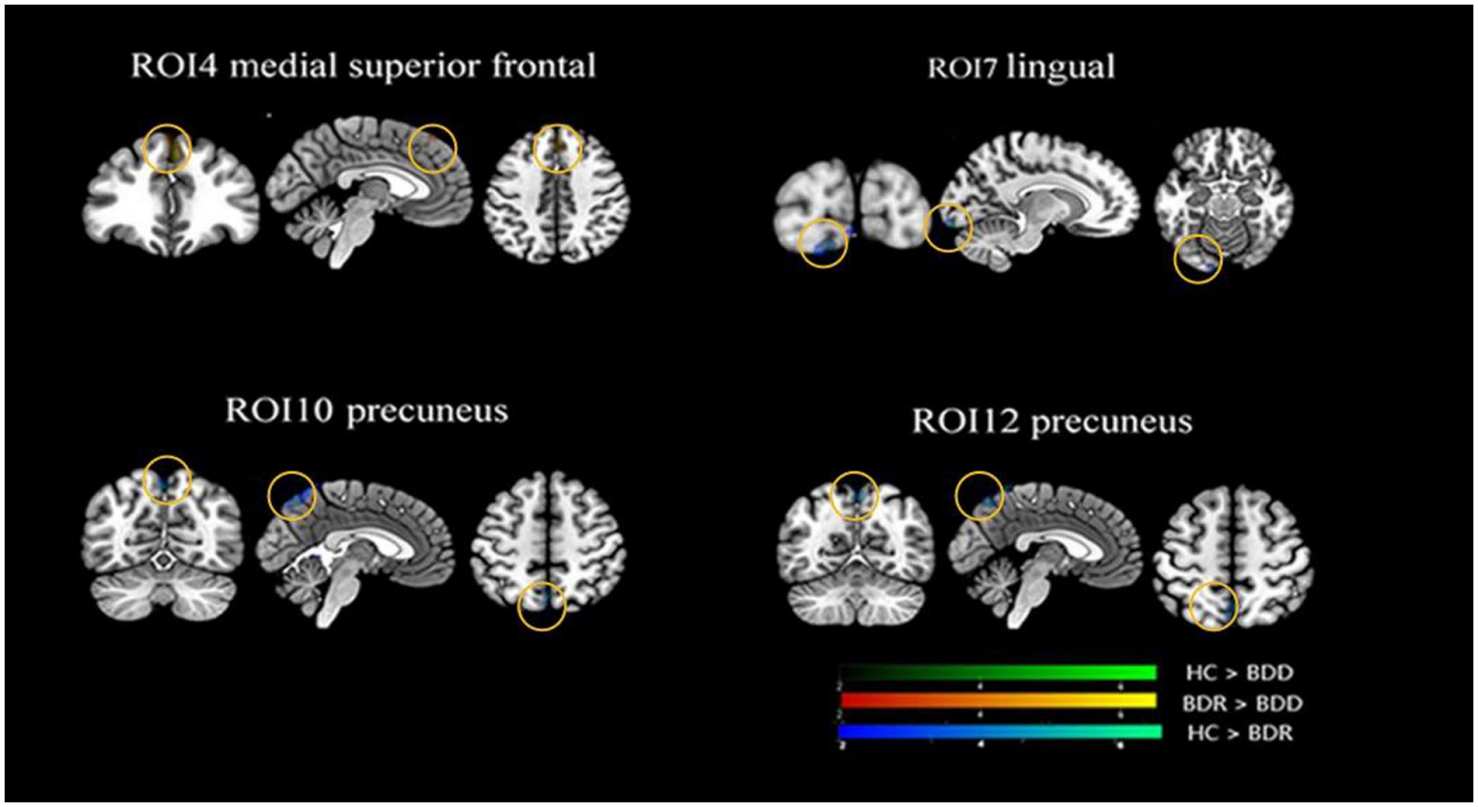
Alternations of functional connectivity in the thalamic-cortical circuit across the BDD, BDR, and HC groups. The ANOVAs for the three groups revealed significant differences in four thalamic subregions cortical connectivity, namely connectivity between the anterior motor thalamus and the medial superior prefrontal, the rostral temporal thalamus and the lingual gyrus, the posterior parietal thalamus and the precuneus/the cerebellum, the occipital thalamus, and the precuneus. Further *post hoc* analysis (P0.05, FWE correction) revealed that the two patient groups had significantly lower functional connectivity between the rostral temporal thalamus and the lingual gyrus, the posterior parietal thalamus, and the precuneus/cerebellum, and the occipital thalamus and the precuneus than the healthy, with no significant difference found between the two patient groups. However, the depressive group, but not the remission group, had significantly lower functional connectivity between the anterior motor thalamus and the medialsuperior frontal cortex than the healthy control. HC, healthy control; BDR, bipolar remission; and BDD, bipolar remission.

Significant differences in cortical connectivity between the medial premotor thalamus and the medial superior prefrontal, the rostral temporal thalamus and the lingual gyrus, the posterior parietal thalamus and the precuneus/the cerebellum, and the occipital thalamus and the precuneus were found in the ANOVAs for the three groups. Further *post hoc* analysis revealed that (*p* < 0.05, FWE correction), the two patient groups had significantly lower functional connectivity between the rostral temporal thalamus and the lingual gyrus, the posterior parietal thalamus and the precuneus/cerebellum, and the occipital thalamus and the precuneus than the healthy, while there was no significant difference between the two patient groups. However, compared to the healthy control, the depressive group had significantly lower functional connectivity between the medial premotor thalamus and the medial superior frontal cortex (see Table 2; Figure 2).

**Table 2:** Pairwise comparison of cortical-thalamic functional connectivity.

Thalamic subregions	Regions	MNI coordinates	Cluster size	*F*	*p*_FWE_	*t* contrast
Medial premotor thalamus (ROI4)	medial superior frontal _L	0, 33, 42	63	17.89	0.012^*^	BDR > BDD
						HC > BDD
Rostral temporal thalamus (ROI7)	lingual _L	−12, −96, −15	250	17.09	0.023^*^	HC > BDR
						HC > BDD
Posterior parietal thalamus (ROI10)	precuneus_L	−3, −69, 54	271	21.39	0.001^**^	HC > BDR
						HC > BDD
	cerebellum_R	6, −45, 3	551	18.79	0.006^*^	HC > BDR
						HC > BDD
Occipital thalamus (ROI12)	precuneus_L	−3, −51, 72	59	16.24	0.044^*^	HC > BDR
						HC > BDD

HC, healthy control; BDR, bipolar remission; BDD, bipolar remission; L, left; R, right; ROI, region of interesting.

### 3.3. Correlation between abnormal functional connectivity and clinical variables

Pearson’s correlation analysis was performed between the BOLD signals showing abnormal functional connectivity across the three groups and the clinical scale and found a significant negative correlation between the mean value of medial superior frontal-medial premotor thalamus connectivity and the score of the HAMD scale (*r* = −0.392, *p* < 0.001).

## 4. Discussion

In this study, a comparison of the connectivity discrepancy in the thalamo-cortical circuit across bipolar depression, remission, and healthy control was made, and it was found that dysconnectivity mainly existed in the prefronto-thalamo-cerebellar and sensorimotor-thalamic circuits. Compared to the healthy controls, the two patient groups showed significantly inferior connectivity in sensorimotor-thalamic; however, patients with depressive episodes not in remission showed significantly lower connectivity in the prefronto-thalamic circuit. In the intervening time, a significantly negative correlation was found between the BOLD signal of the prefronto-thalamic and the score of the HAMD scale.

The medial premotor thalamus comprises the ventral anterior nucleus (VA) and ventrolateral nucleus (VL), which project to the premotor cortices. Our study revealed that there was decreased connectivity between the medial premotor thalamus and the medial superior frontal cortex in patients with bipolar depression. Previous studies have primarily concentrated on the functional disruption of higher-order thalamic nuclei in bipolar disorder, such as the dorsomedial nucleus (15). However, our findings indicate that the first-order thalamic nucleus also plays a direct role in the cortical-thalamic circuit. The prefronto-thalamic circuit is widely considered as a cognitive-related circuit, playing a vital role in execution (24–26). Disrupting the circuit may cause damage to the regulation of emotion and cognition reactions. The medial superior frontal cortex showed abnormal connectivity in our study, which outputs signals to cortex and subcortical regions (27), such as the posterior cingulate, thalamus, amygdala, and hippocampus, function as top-down regulation on attention bias, behavior inhibition and working memory (28, 29). For bipolar depressive patients, weakened connectivity in the prefronto-thalamic subregions may associate with patients’ disruption in the working memory maintenance and social interaction (30, 31).

Numerous studies have demonstrated that individuals with depression exhibit impaired functional connectivity of prefronto-thalamic circuitry compared to healthy controls (32, 33). For instance, Zhang et al. (32) discovered that the group of patients with bipolar depression displayed significantly lower functional connectivity between the bilateral thalamus and frontal cortex during the resting state. In this study, a significant positive correlation was noted between the HAMD score and the BOLD signal of the prefronto-thalamus, which also demonstrated that enhancing prefronto-thalamus connectivity may help to relieve depression. The prefronto-thalamo hypoconnectivity was particularly evident in patients with depression but not in those in remission, suggesting that the distinct state characteristic between the two states is potentially located in the prefronto-thalamic circuitry.

Both patient groups showed significantly weakened connectivity between the posterior parietal thalamus, which primarily encompasses the anterior portion of the pulvinar, and the cerebellum. The pulvinar, which is the largest nucleus of the thalamus, is largely connected to the anterior cingulate and prefrontal areas (34). And the cerebellum is widely believed to be involved in motor function (35, 36), cerebellar lesion can lead to motor function impaired. In this study, both the patients groups showed hypoconnectivity between the thalamus and cerebellar, which may contribute to bipolar patients’ psychomotor abnormalities such as psychological retardation and impulsivity. Researches have also confirmed that abnormal psychomotor of mental disorder is associated with dysfunction of the cerebellum and thalamus (37). Furthermore, the cerebellum projects to several regions of the prefrontal cortex through the thalamus, including the medial, dorsal, and lateral areas of the prefrontal region (38). And one crucial study have confirmed that, the prefronto-thalamo-cerebellar circuitry is closely related to motor plan (39), persistent information expression of the frontal during motor planning is dependent on the cerebellum, therefore, the disruption of this circuitry may suggest abnormal psychomotor.

Besides, brain damage studies in mice have found that cerebellum damaged mice suffered from spatial and working memory disruption. For instance, Lalonde (40) observed that cerebellum damaged mice run a maze, and found that cerebellar lesions would cause cognitive deficits resulting in impaired completion of maze tasks. Therefore, it is likely that the diminished functional connectivity of the prefronto-thalamo-cerebellar circuitry may be linked to impairments in working memory, planning, and rule-based learning among patients.

Both patient groups exhibited hypoconnectivity in the sensorimotor-thalamic circuit, which includes the precuneus and lingual regions, consistent with prior research (33). Historically, the precuneus has received little scrutiny, possibly due to its concealed location and the lack of studies on focal lesions (41). However, recent studies have indicated that the precuneus is an integral component of the network of neural correlates of consciousness (42, 43), which may contribute to alterations in conscious states, such as sleep. Therefore, it is plausible that the weakened connectivity between the precuneus and thalamus could be associated with sleep disturbances in patients.

A few limitations should be noted in this study. First, medication may be a confounding factor. The majority of the recruited patients were on medications; most of them were consuming mood stabilizers and other medications such as antipsychotics, antidepressants, or benzodiazepines, depending upon the patient’s clinical performance. Additional studies in drug-naive BD patients are crucial to confirm the findings of this study. Second, the study is a cross-sectional design, which limits the assessment of the dynamic changes between depression and remission in BD patients; thus, future longitudinal studies with drug-naive patients are required to clarify the dynamic transformation.

## 5. Conclusion

In this study, both patient groups displayed abnormality in the prefronto-thalamic-cerebellar and sensorimotor-thalamic circuits, depressive patients specifically exhibited decreased connectivity in the prefronto-thalamic circuit, which perhaps was associated with their cognitive disruption and rumination thinking. And remission patients also exhibited weakened connectivity in the sensorimotor-thalamic circuit; however, they significantly increased the connectivity of prefronto-thalamic compared to the depressive group. The results showed that the irregularity of the sensorimotor-thalamic circuit possibly is the trait-related distinguishing factor of bipolar disorder, while the prefronto-thalamic circuit is the state-related characteristic feature.

## Data availability statement

The original contributions presented in the study are included in the article/supplementary material; further inquiries can be directed to the corresponding author.

## Ethics statement

The studies involving human participants were reviewed and approved by ethics committee of Xiangya Second Hospital. The patients/participants provided their written informed consent to participate in this study.

## Author contributions

CZ wrote the main manuscript text. SL edited language. WP designed the experiments. All authors contributed to the article and approved the submitted version.

## Conflict of interest

The authors declare that the research was conducted in the absence of any commercial or financial relationships that could be construed as a potential conflict of interest.

## Publisher’s note

All claims expressed in this article are solely those of the authors and do not necessarily represent those of their affiliated organizations, or those of the publisher, the editors and the reviewers. Any product that may be evaluated in this article, or claim that may be made by its manufacturer, is not guaranteed or endorsed by the publisher.

## Footnotes

^1^http://atlas.brainnetome.org/index.html

^2^http://www.fil.ion.ucl.ac.uk/spm
